# Supplementary material for: Acanthamoeba castellanii Genotype T4: Inhibition of Proteases Activity and Cytopathic Effect by Bovine Apo-Lactoferrin
Source: Microorganisms. 2023 Mar 9;11(3):708. doi: 10.3390/microorganisms11030708 (PMC10059889; doi:10.3390/microorganisms11030708)
Supplement: Supplementary file 1 [file microorganisms-11-00708-s001.zip › microorganisms-2202097-supplementary.pdf]

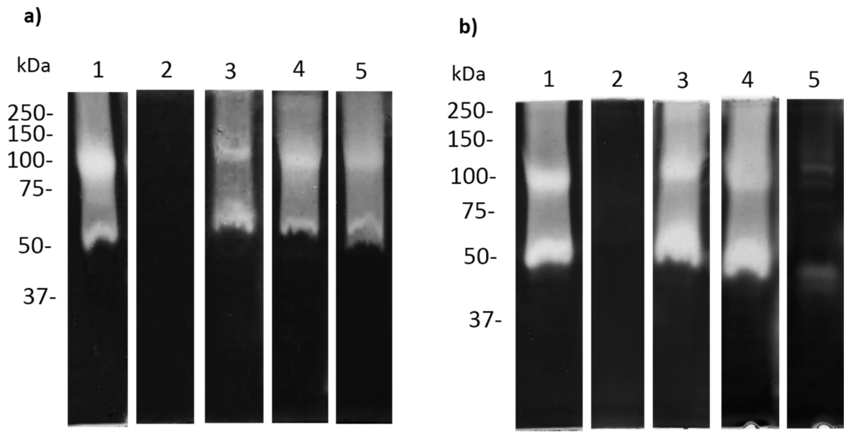

Figure S1. Inhibition of human apo-Lf proteases from TCE and CM from *A. castellanii*. Zymography using 10% SDS-PAGE co-polymerized with 0.1% apo-hLf. Samples of TCE (**a**) and CM (**b**) from *A. castellanii* trophozoites were incubated for 1 h with different protease inhibitors: pHMB (lane 2), E-64 (lane 3), PMSF (lane 4), and aprotinin (lane 5). Untreated *A. castellanii* trophozoites without inhibitors were used as the experimental control (**a**, **b**) lanes 1). The gels were activated overnight at 37 °C and pH 7.0. Three independent assays were performed.
